# The Re-Education of the Defective

**Published:** 1918-06-01

**Authors:** 


### PHYSICAL CULTURE IN EDUCATION.

### III. The Re-education of the Defective.

In the previous papers of this series we have considered the place of physical exercises in the development of the normal, healthy individual. In this we propose to deal briefly with the applications of exercise in an undeveloped or pathologic condition of mind and body.

The training of mentally defective children has received increasing attention of late years, a sign, doubtless, of the rapid awakening of a social conscience in legislation. How pressing a matter it has become is revealed by the statistics of Dr. Francis Warner, who in the examination of 100,000 London school children found over 1 per cent. actual mental defectives, and in America similar investigations show a corresponding proportion. The symptoms of deficiency are characteristic. The child is smaller than its fellows, heavy in expression, dull and listless, often exhibiting the adenoid facies. He is idle, unruly, stubborn, and slow to discriminate differences of weight, colour, etc. The most marked feature in the majority of cases is a lack of co-ordination, the child cannot copy simple figures or write with facility, and gymnastic exercises are accomplished without dexterity and skill. It is by perfecting or improving the neuro-muscular apparatus that these cases—generally the children of the poor—are put in the way of gaining a livelihood.

### Actual Deficiency or Retarded Development.

The most reliable method of distinguishing between actual deficiency and a retarded development, due to unfavourable surroundings, ill-health, or insufficient food, is by means of the Binet-Simon scale in one of its modifications. Every child who is more than a year behind his grade should be examined, unless sufficient cause be found from purely physical causes to account for the delay.

When such a weeding-out has been accomplished the actual dullards are placed in special classes, graded according to the standard of the child's intelligence. The co-operation of the parent is in all cases invited, but care has to be taken not to arouse the hostility of the parent by telling her that her child is an idiot, etc. In America special child-study committees undertake this task, and such an institution would be of great value over here. In London there are at the present time over sixty classes where defective children are trained in handicrafts by which they will in later years be able to gain a livelihood, and in most other European countries similar schemes are in operation.

### The Training of Backward Children.

This must proceed along certain well-defined lines. Physical condition is the first care, for until the body is well nourished the brain will be unable to respond adequately to impressions conveyed to it. The mental improvement resulting from sufficient food, exercise, sleep, and strict attention to bodily functions is often striking.

The next stage is that of opening up the obstructed avenues of approach. The limbs must be trained to respond rapidly and accurately, not only because co-ordination in itself is of great value, but because a mind that can command the body accurately is in a better state as regards intelligence, concentration, and observation than one which does not.

### Preliminary Steps.

A start should be made with the simple games of childhood. Running, catching and throwing the ball, jumping, etc., are all exercises calling for co-ordination in large muscle groups. Marching and performing simple evolutions with the accompaniment of music are especially suitable for defective children, who often show an extremely keen sense of rhythm.

When some progress has been made along these lines an effort must be made to secure the finer shades of co-ordination in the muscles of the forearm and hand, and gradually such occupations as basketry and weaving are introduced. But progress is slow, and, though some may by dint of constant supervision become sufficiently advanced to return to the normal schools, the majority require care and supervision throughout their lives.

### Varieties of Physical Methods.

The more educational movements are directed, firstly, towards improving the gait and carriage—the shambling, shuffling gait of the defective child is typical. This is achieved by such devices as running contests on tiptoe, walking with a book on the head, or upon bricks placed at equal intervals, or between the rungs of a ladder. Military drill is also largely employed, its effectiveness consisting in the spirit of emulation to attain the power of others, and in the call for quick and certain response to the word of command. Hand in hand with compulsory drills and exercises go voluntary sports, but the child must have undergone some preliminary training before these can be indulged in with any effect, for lack of initiative is the outstanding feature of the defective child.

### The Child Criminal.

Another type of defective, and one who presents problems of at least as great importance, is the youthful criminal; the hooligan pilferer who appears so frequently before the police-court magistrates. A large percentage of these youthful delinquents are unsound both physically and morally. Their intellects are blunted, often from environmental causes, they are easily led, and in the vicious circle of street, public-house, and jail rapidly deteriorate into the habitual and hopeless criminal.

Here the remedial training must be more Spartan in character. Exercise, as Sargent has pointed out, does not need to be agreeable in order to be beneficial, and military evolutions on the German model have been found in America to yield highly satisfactory results. Here, again, however, the influence of competitive games and sports is all for the good, though a strict supervision is necessary in order to check the tendency to unsportsmanlike behaviour naturally to be expected from their deficient moral sense.

### A Word to Penal Institutions.

In this connection the opinion of Dr. Wey, the head of the Elmira State Penitentiary, U.S.A., and the introducer of many changes in reformatory treatment, is of interest. He says: "There is a class of youthful felons who can be reached in their growth period and improved primarily through the training of the body, the cultivation of the head following in good time. If penal insti-

☛ Previous articles appeared Jan. 12 and March 30, p. 553.

tutions could more often look upon bodily training as a powerful agent for the physical, mental, and moral reformation of their charges, more men would be released at the expiration of their time competent to maintain themselves honestly."

It would be difficult to find a more sweeping indictment of our own penal system. As an example, let us take the case of a class of forty-three dullards who were subjected at Elmira to a course of physical training over a period of sixteen months. This included baths at frequent intervals, in conjunction with passive exercises by a trained masseur, and a class in calisthenics. They were placed upon a specially nourishing diet, and, as they grew more efficient, exercises of increasing complexity were introduced. The physical training lasted two hours daily, and they were excused work in the shops, though their schooling continued as usual.

At the end of six months the improvement was marked. There was an average gain in weight of one and a quarter pounds, the whole expression of the face had changed, the dull, stolid look assumed a more intelligent expression, the eye gained in brightness and vivacity. Their average marking in school rose from 45.2 per cent. to 74.16—a truly striking increase.

If such results can be obtained in the United States there seems to be no reason why our own prison authorities should not make some effort to transform our jails into places for the re-moulding of character instead of being content to regard them as grim repositories for the storage of society's derelicts. The reform of social conditions and the reform of the criminal, one so largely the product of the other, must necessarily proceed hand in hand.

